# Problematic Gambling Behavior in a Sample with Substance Use Disorder: The Role of Attachment Style and Alexithymia

**DOI:** 10.1007/s10899-022-10154-2

**Published:** 2022-09-24

**Authors:** Mª Begoña Rueda Ruiz, Urko Aguirre Larracoechea, Marta Herrero, Ana Estévez

**Affiliations:** 1 Psychiatry Service of the Barrualde-Galdakao Hospital, Galdakao Hospital, Labeaga Auzoa 46A - 48960, Galdakao, Bizkaia, Spain; 2 Faculty of Health Sciences, Deusto University, Bilbao, Spain

**Keywords:** gambling disorder, substance use disorder, comorbidity, attachment, alexithymia

## Abstract

Gambling disorder is a high comorbid disorder in substance abusers which conjunct appearance is related to worse symptomatology and evolution. Nevertheless, the research on the risk factors that may explain this comorbidity is scarce. We build of the self-regulation theory of attachment and addiction to examine if insecure attachment is related to gambling comorbidity in substance abuse disorder and the mediating role of alexithymia in this process. A cross-sectional study was carried out with 369 clinical patients with substance use disorder of which 69 presented comorbid gambling disorder diagnosed with the DSM-5 criteria. Results showed that insecure attachment was more prevalent in the group with comorbid gambling. In this group, the alexithymia levels were also higher and mediated the relationship in between attachment and gambling disorder comorbidity even controlling for several sociodemographic variables. This research indicates that insecure attachment enhances the risk of gambling comorbidity on substance abusers due to the detrimental effect on the self-regulation of emotion. Thus, interventions directed to increase the identification, expression and awareness of emotions might help to reduce comorbidity of gambling of substance use disorders.

**Supplementary Information:**

The online version contains supplementary material available at 10.1007/s10899-022-10154-2.

## Introduction

Research on behavioral addictions has, in recent years, significantly increased. This is due to changing assumptions regarding how people can become dependent not only on psychoactive substances but also on specific behaviors (Zilberman et al., 2018). Among behavioral addictions, gambling has been included as an addictive disorder since 2013 when it was included in the fifth edition of the Diagnostic and Statistical Manual (Goudriaan, Brink, & Holst, 2019). In fact, “substance abuse” is the term with the greatest presence in gambling-related research with young people due to this new categorization of addictions (Yalçin, 2022). Gambling disorder is described as persistent gambling behaviors that an individual is unable to control despite negative psychosocial and financial consequences (APA, 2013).

The prevalence of gambling disorder in the general population is approximately 0.5%, indicating that 5 out of 1000 people may experience a gambling disorder in their lifetime (Potenza et al., 2019). However, this prevalence increases to 14-23% in individuals with substance use treatment (Cowlishaw et al., 2014) and up to 28-50% in individuals with substance use disorder (Grant & Chamberlain, 2020), suggesting that gambling may be an important comorbid addiction in substance use disorders. Some studies have suggested that the co-occurrence of gambling problems with substance abuse in individuals may be related to more severe psychological symptoms (Abdollahnejad et al., 2014; Langenbucher & Merrill, 2001) than substance abuse behaviors alone (Langenbucher & Merrill, 2001).

Despite the high prevalence of problematic gambling in conjunction with substance use disorders and its potential health impact, the research on this association is scarce (Cowlishaw et al., 2014; Estévez et al., 2021; Petry, 2001; Rash et al., 2016) and has been developed primarily using general populations or community samples (Abdollahnejad et al., 2014; Barnes et al., 2015; El-Guebaly et al., 2006). However, the prevalence of gambling in clinical samples of individuals seeking treatment for substance abuse might be significantly higher (Hodgins & El-Guebaly, 2010). Furthermore, the factors involved in the co-occurrence of gambling with substance abuse may differ from those in non-clinical samples (Estévez et al., 2021). Therefore, to promote better interventions for both addictions, more studies are needed to understand the vulnerability factors involved in the appearance of gambling problems in individuals with substance use disorder (Rash et al., 2016).

The high comorbidity between gambling disorder and substance abuse may be explained by common etiological and maintenance factors (Goudriaan et al., 2019; Mallorquí-Bagué et al., 2016), among which the self-regulation model proposes a common origin based on attachment trauma (Lafond Padykula & Conklin, 2010). According to this model, attachment figures serve as models for the control and management of behavior. In cases of impaired attachment, an individual does not adequately learn how to manage his/her emotions and impulses. The affective regulatory characteristics by which insecure attachment has a detrimental effect may, therefore, explain addictive behaviors (Nakhoul et al., 2020; Toqeer et al., 2021). Insecure attachment promotes inadequate affective regulation, which may lead to lower consciousness, comprehension, and management of emotions. This reduced emotional regulatory ability increases the probability of addiction as these behaviors are an opportunity to reduce the psychological distress of not understanding and adequately handling feelings (Fletcher et al., 2015; Lafond Padykula & Conklin, 2010; Mestre-Bach et al., 2020).

Several studies support the importance of attachment in substance use disorders (Fairbairn et al., 2018; Schindler, 2019; Schindler & Bröning, 2015). In a meta-analysis by Fairbain et al. (2018), the results of 34 longitudinal studies (with a total of 56,721 participants) found that insecure attachment was a significant risk factor for substance addiction. Moreover, while the literature on gambling disorder is not extensive, it does highlight how insecure attachment is an important risk factor for several gambling indicators (Estévez et al., 2020; Estevez et al., 2019; Jauregui & Estevez, 2020; Kaya & Deveci, 2021; Keough et al., 2018).

Alexithymia is an affective regulatory characteristic that may explain the effects of attachment. It is a personality trait whereby an individual has difficulty identifying and expressing emotions, and being aware of the feelings and emotions others and one’s self (Luminet et al., 2018). Alexithymia is strongly influenced by emotional learning from attachment figures. Hence, the lack of adequate emotional regulation modeling and suitable responses to the feelings of children can lead to difficulties in acquiring functional regulation skills (Lafond Padykula & Conklin, 2010; Luminet et al., 2018).

The literature on the relationship between alexithymia and addiction has uncovered that difficulties in emotional regulation is highly prevalent in individuals with substance use disorders (Morie et al., 2016) and are more frequent in those at risk of gambling addiction (Estévez et al., 2020, 2022; Marchetti et al., 2019). In addition, empirical results have shown that alexithymia may serve as an explanatory mechanism for the effects of insecure attachment on different addictions, including substance abuse and gambling disorders (Estévez et al., 2020; Sung et al., 2020; Toqeer et al., 2021; Zdankiewicz-Scigała & Scigala, 2020). Recent studies have also suggested that alexithymia mediates the relationship between insecure attachment and the risk of gambling disorder in clinical and general population samples (Di Trani et al., 2017; Gori et al., 2022). However, despite these results, the literature on the mediating role of alexithymia remains scarce (Estévez et al., 2020).

Based on the self-regulation model, the co-occurrence of multiple addictions could be explained by attachment styles that limit regulatory processes (Lafond Padykula & Conklin, 2010). The available literature suggests that individuals with substance use disorders typically show more insecure attachment styles (Hiebler-Ragger & Unterrainer, 2019) and, in cases of individuals with substance abuse disorder and gambling, more emotional regulatory problems (Langenbucher & Merrill, 2001; Petry, 2001, 2007). Unfortunately, there are few studies that have investigated differential attachment styles in persons with substance use disorder and other addition comorbidities (Hiebler-Ragger & Unterrainer, 2019) and, to our knowledge, none that have compared the prevalence of insecure attachment in individuals with substances use disorders and problematic gambling behaviors and the potential role of alexithymia in this relationship.

Therefore, this study investigated the presence of problematic gambling behaviors in a clinical sample of individuals with substance use disorder and aimed to ascertain whether impaired attachment (specifically alexithymia) is a risk factor for gambling addiction. We hypothesized that there would be a significant subsample with problematic gambling behaviors among individuals with substance use disorder and that there would be a higher prevalence of insecure attachment in this group (who would score higher in alexithymia) than those without a gambling addiction. Alexithymia was also expected to act as a mediator that explains the relationship between insecure attachment and problematic gambling behavior.

## Methods

### Participants

In this study, there were 369 **spanish** participants with substance use disorders recruited from a hospital dual diagnosis unit. Exclusion criteria for sample were to have brain damage, intelectual disability or limitations to understand the ítems of the used tests. Problematic gambling behavior was present in 26.29% (n = 97) of the participants.

### Instruments

#### Sociodemographic Variables, Psychological Disorders and Poly-Consumption

Sociodemographic variables including age (Jiménez-Murcia et al., 2014; Savvidou et al., 2017), sex (Kessler et al., 2008; Parhami et al., 2014), marital status (Subramaniam et al., 2015), and educational level (del Pino-Gutiérrez et al., 2017; El-Guebaly et al., 2006) have been shown to be related to addictions and their severity. In addition, there is a strong association between addictions and psychological disorders (Parhami et al., 2014; Rash et al., 2016), and the presence of mental health issues in individuals with substance abuse disorder may affect the severity of gambling behaviors (El-Guebaly et al., 2006). Poly-consumption, which refers to the abuse of multiple addictive substances, is also related to severe addictive patterns and poor intervention outcomes (Jeffirs et al., 2019; Wu et al., 2011). Therefore, these variables were also taken into account when gathering information on risk correlates and to isolate the effects of attachment and alexithymia.

#### Adult Attachment Style

Both insecure and secure attachment styles were assessed using the Questionnaire of Adult Attachment by Melero & Cantero (2008). This 40-item scale evaluates four affective-relational dimensions: (1) Low self-esteem, the need for approval, and fear of rejection; (2) hostile conflict resolution, resentment, and possessiveness; (3) the expression of feelings and comfort with relationships; and (4) emotional self-sufficiency and discomfort with intimacy. The items were assessed using a 6-point Likert scale from 1 (completely disagree) to 6 (completely agree). The two attachment styles (secure and insecure) were evaluated based on the scores obtained from measuring the four dimensions. Participants were considered to have secure attachments with scores lower than 28.54, 22.34 and 13.99 in the low self-esteem, hostile conflict resolution, and emotional self-sufficiency dimensions, respectively and a score above 44.13 in the expression of feelings dimension. All other scores were considered to represent insecure attachment. The validity of the scale has been observed as the proposed attachment styles differed in the expected underlying constructs based on the attachment theory (Melero & Cantero, 2008). The average reliability of the scales was good in the original sample (α = 0.78), and in this research it was α = 0.77; ω = 0.83.

#### Alexithymia

Alexithymia was measured using the Spanish version (Martínez Sanchez, 1996) of the Toronto Alexithymia Scale-20 (TAS-20; Bagby et al., 1994). This questionnaire comprises 20 items assessed on a 6-point Likert scale ranging from 0 (strongly disagree) to 5 (strongly agree) and evaluates alexithymia using global dimensions that include: (1) difficulty identifying emotions and differentiating them from body sensations; (2) difficulty describing feelings to others; and (3) an outwardly positioned style of thinking. This questionnaire has a good discriminant validity as it discerns adequately between psychiatric patients and non-patients (Kooiman et al., 2002). The scale has good average internal consistency (α = 0.83) and in the present study it was α = 0.74; ω = 0.82.

#### Problematic Gambling Behavior

Problematic gambling behavior was measured using the *Cuestionario Breve de Juego Patológico* [Brief Questionnaire of Pathological Gambling] developed by Fernández-Montalvo et al., (1995). This is a screening tool consisting of four items measured using a ‘yes/no’ dichotomous scale. The items measure whether an individual perceives themselves to have gambling problems, if they feel guilty about their gambling, if they are unable to stop gambling despite the desire to do so, and whether they have debts or take money from home to gamble. Problematic gambling behavior was considered to be present when the individual answered positively to at least one of the four questions. The sensitivity of the tool is 100%, the specificity is 97.5%, and diagnostic efficacy is 97.88% which indicates adequate discriminant validity. As well, the measure shows high convergent validity with other gambling questionnaires and the presence of clinical gambling diagnosis. The internal consistency of the scale is 0.94, and the test-retest reliability is 0.99, which indicates adequate reliability indexes (Fernández-Montalvo et al., 1995).

#### Psychological Symptoms

Mental health was assessed using the Symptoms Check List 90-R (SCL 90-R; Derogatis 2002; Spanish version of González de Rivera et al., (2002). Study participants answered questions that assessed their experience of different symptoms in the previous seven days using a 5-point Likert scale that ranged from 0 (not at all) to 4 (completely). The 90-item questionnaire measured nine clinical subscales: depression, anxiety, somatization, obsessive-compulsion, interpersonal sensitivity, phobic anxiety, psychoticism, paranoid ideation, and hostility. The questionnaire also provides a composite indicator of mental health called the global severity index. The reliability of the dimensions ranges from 0.77 to 0.90 and, in this study, it was α = ω = 0.93. The questionnaire has adequate concurrent and discriminant validity that indicate that the scale is useful to identify the assessed symptomatology as well that to differentiate in between clinical and non-clinical populations (González de Rivera et al., 2022).

### Procedure

Participants were a convenient sample recruited by the same psychologist during their stay at the dual diagnosis unit. All participants received clinical treatment for the consumption of at least one psychoactive substance other than tobacco. They fill out all of the questionnaires at one time, five days after their arrival at the hospital.

The study procedures were carried out in accordance with the Declaration of Helsinki and was approved by the Ethics and Research Committee of the Hospital (Project 23/17). All participants were given information about the study and provided informed consent. No incentive was provided for study participation.

### Statistical Analysis

All statistical procedures were conducted using SAS for Windows, version 9.4 (SAS Institute, Cary, NC). A p-value < 0.05 was considered significant unless otherwise stated. An exploratory data analysis was performed to describe demographic, clinical, and psychological features based on the presence of problematic gambling and an insecure attachment style. The frequency and percentage of each category of categorical variables and mean and standard deviation of continuous variables was computed. The nonparametric Wilcoxon (continuous variables) and the Chi-square tests (Fisher’s Exact test when needed) were applied. A multivariate logistic regression analysis was then developed to identify predictor factors that could influence problematic gambling. Exposure variables with p-values < 0.20 (Vittinghoff et al., 2012) in the exploratory analysis were included in the regression model as independent variables. Using a backward procedure, a final multivariate logistic regression model was obtained. Model effects were expressed using odds ratios (ORs) and 95% confidence intervals (CIs). Model robustness was assessed in terms of discrimination and calibration. Discrimination, defined as the ability to distinguish events from non-events, was evaluated by computing the area under the receiver operating characteristic (ROC) curve. If the model had an area under the ROC curve (AUC) value > 0.70 it was considered well discriminated (Hosmer et al., 2015). The AUC was obtained by bootstrapping 2000 samples. Calibration was determined using the Hosmer-Lemeshow test to gauge the goodness-of-fit capacity of the model. A p-value > 0.05 indicated good calibration (Hosmer et al., 2015). The effect of insecure attachment on problematic gambling behaviors as mediated by alexithymia was examined using 1000 bootstrapped samples.

## Results

### Sociodemographic and Psychological Differences between Participants with and without Problematic Gambling Behaviors

All study participants were over 18 years of age, with an average age of 44.14 years (SD = 10.40). Most participants were men (68.56%). A majority of the participants had finished primary school (37.67%), followed by professional training (28.18%), and secondary school (18.4%). Less common were university graduates (9.76%) or those with no formal schooling (5.96%). Approximately half of the participants were single (50.41%), 13.55% were married, 23.58% were divorced/separated, 9.49% had legal partners, and 2.98% were widowed. Workers accounted for 37.67% of the sample, 3.52% were retired, and 2.44% were students. The majority were unemployed (40.38%), with 2.71% unable to work.

A majority of the participants (78.86%) used more than one substance (excluding tobacco), 13.55% consumed only alcohol, 2.71% used fentanyl, and 4.88% used other substances. Most of the sample (80.49%) had previously been in psychological treatment for substance abuse while 6.78% had previously sought psychological treatment for gambling.

As shown in Table 1, men had a 3.93 times higher probability of having problematic gambling behaviors than women. Single participants had a lower probability of gambling, with nearly two and a half times less risk of gambling compared to those who had a different marital status. This was especially evident in widows, who were 2.32 times more likely to exhibit problematic gambling behavior. Having previously attended treatment for substance abuse was related to a lower odds of gambling problems compared to those who had never attended a treatment program (OR = 0.55). However, those who had previously received psychological treatment specifically for gambling were 13.87 times more likely to have problematic gambling behaviors. Individuals who abused multiple types of substances were two and a half times more likely to have problematic gambling behaviors. There were no differences in gambling behavior by educational level, employment status, tobacco use, or age.

**Table 1:** Differences in participant sociodemographic characteristics by the co-occurrence of problematic gambling and substance abuse

			Problematic Gambling						
	Total (n = 369)		No (n = 272)		Yes (n = 97)				
Variable	n	(%)	n	(%)	n	(%)	χ^2^	*df* ^*a*^	*p*
Sex							19.86	1	< 0.001
Men	253	(68.56)	169	(66.80)	84	(33.20)			
Women	116	(31.44)	103	(88.79)	13	(11.21)			
Age							0.72	3	0.860
18–25	19	(5.16)	15	(78.95)	4	(21.05)			
26–40	105	(28.53)	75	(71.43)	30	(28.57)			
41–55	202	(54.89)	151	(74.75)	51	(25.25)			
≥ 56	42	(11.41)	30	(71.43)	12	(28.57)			
Educational level							8.30	4	0.081
No formal education	186	(50.41)	126	(67.74)	60	(32.26)			
Primary school	50	(13.55)	41	(82.00)	9	(18.00)			
Secondary school	35	(9.49)	27	(77.14)	8	(22.86)			
Professional training	87	(23.58)	67	(77.01)	20	(22.99)			
University studies	11	(2.98)	11	(100.00)	0	(0.00)			
Marital status							9.81	4	0.044
Single	22	(5.96)	12	(54.55)	10	(45.45)			
Married	139	(37.67)	98	(70.50)	41	(29.50)			
Legal partner	68	(18.43)	52	(76.47)	16	(23.53)			
Divorced/ separated	104	(28.18)	79	(75.96)	25	(24.04)			
Widowed	36	(9.76)	31	(86.11)	5	(13.89)			
Employment							2.67	5	0.750
Worker	139	(37.67)	107	(76.98)	32	(23.02)			
Student	9	(2.44)	7	(77.78)	2	(2.22)			
Unemployed	149	(40.38)	107	(71.81)	42	(28.19)			
Retired	13	(3.52)	10	(76.92)	3	(23.08)			
Unable to work	59	(15.99)	41	(69.49)	18	(30.51)			
Previous psychological treatment for substance abuse							4.46	1	0.038
No	72	(19.51)	46	(63.89)	26	(36.11)			
Yes	297	(80.49)	226	(76.09)	71	(23.91)			
Previous psychological treatment for gambling							39.93	1	< 0.001
No	344	(93.22)	267	(77.62)	77	(22.38)			
Yes	25	(6.78)	5	(20.00)	20	(80.00)			
Tobacco use							0.16	1	0.863
No	61	(16.53)	50	(81.97)	11	(18.03)			
Yes	308	(83.47)	222	(72.08)	86	(27.92)			
Poly-consumption (excluding tobacco)							6.07	1	< 0.001
No	81	(21.95)	69	(83.78)	12	(16.22)			
Yes	288	(78.05)	203	(70.49)	85	(29.51)			

^*a*^*df*, degrees of freedom

The alexithymia and the psychological symptomatology results showed statistically significant differences between attachment styles, with insecure attachment related to an increased probability of gambling problems (see Table 2).

**Table 2:** Differences in psychological variables by the co-occurrence of problematic gambling with substance abuse

			Problematic Gambling					
	Total (n = 369)		No (n = 272)		Yes (n = 97)			
Variable	n	(%)	n	(%)	n	(%)	*χ* ^*2*^	*p*
Attachment style							7.99	0.005
Secure attachment	131	(35.50)	108	(82.44)	23	(17.56)		
Insecure attachment	238	(64.50)	164	(68.91)	74	(68.91)		
Variable	*M* ^*a*^	*SD* ^*b*^	*M*	*SD*	*M*	*SD*	*TW* ^*c*^	*p*
Alexithymia	59.75	10.87	58.94	11.04	62.02	10.41	2.41	0.016
Global severity index	1.80	0.72	1.78	0.72	1.88	0.72	1.10	0.271
Somatization	1.77	0.90	1.78	0.92	1.75	0.85	-0.13	0.895
Obsession-Compulsion	1.95	0.86	1.91	0.87	2.06	0.82	1.50	0.133
Interpersonal Sensitivity	1.65	0.89	1.61	0.88	1.78	0.91	1.37	0.169
Depression	2.29	0.85	2.28	0.86	2.38	0.83	0.14	0.886
Anxiety	1.84	0.90	1.81	0.92	1.93	0.86	1.13	0.258
Hostility	1.58	1.03	1.52	1.04	1.74	0.98	2.06	0.039
Phobic Anxiety	1.12	0.91	1.10	0.90	1.17	0.94	0.68	0.499
Paranoid Ideation	1.73	0.92	1.66	0.92	1.93	0.91	2.40	0.017
Psychoticism	1.56	0.80	1.52	0.79	1.67	0.84	1.46	0.145

^a^M, median

^b^SD, standard deviation

^c^TW, Wilcoxon non-parametric test

Higher alexithymia scores were related to a greater likelihood of problematic gambling. Overall, the psychological variables were not related to the co-occurrence of problematic gambling in participants with substance use disorders as indicated by the scores on the global severity index and most of the psychological symptomatology (i.e., somatization, obsessive-compulsion, interpersonal sensitivity, depression, anxiety, phobic anxiety, and psychoticism). However, hostility and paranoid ideation were both related to higher odds of gambling problems.

**Table 3:** Differences in participant sociodemographic characteristics by attachment style

			Insecure attachment style						
	Total (n = 369)		No (n = 131)		Yes (n = 238)				
Variable	n	(%)	n	(%)	n	(%)	χ^2^	*df* ^*a*^	*p*
Sex							0.47	1	0.910
Men	253	(68.56)	89	(67.94)	164	(68.91)			
Women	116	(31.44)	42	(32.06)	74	(31.09)			
Age							7.22	3	0.065
18–25	19	(5.16)	5	(3.85)	14	(5.88)			
26–40	105	(28.53)	40	(30.77)	65	(27.31)			
41–55	202	(54.89)	78	(60.00)	124	(52.10)			
≥ 56	42	(11.41)	7	(5.38)	35	(14.71)			
Educational level							4.08	4	0.395
No formal education	186	(50.41)	9	(6.87)	13	(5.46)			
Primary school	50	(13.55)	49	(37.40)	90	(37.82)			
Secondary school	35	(9.49)	27	(20.61)	41	(17.23)			
Professional training	87	(23.58)	30	(22.90)	74	(31.09)			
University studies	11	(2.98)	16	(12.21)	20	(8.40)			
Marital status							9.40	4	0.052
Single	22	(5.96)	62	(47.33)	124	(52.10)			
Married	139	(37.67)	18	(13.74)	32	(13.45)			
Legal partner	68	(18.43)	16	(12.21)	19	(7.98)			
Divorced/ separated	104	(28.18)	27	(20.61)	60	(25.21)			
Widowed	36	(9.76)	8	(6.11)	3	(1.26)			
Employment							9.51	5	0.091
Employed	139	(37.67)	62	(47.33)	77	(32.35)			
Student	9	(2.44)	3	(2.29)	6	(2.52)			
Unemployed	149	(40.38)	42	(32.06)	107	(44.96)			
Retired	13	(3.52)	3	(2.29)	10	(4.20)			
Unable to work	59	(15.99)	21	(16.03)	38	(15.97)			
Previous psychological treatment for substance abuse							0.89	1	0.345
No	72	(19.51)	29	(22.14)	43	(18.07)			
Yes	297	(80.49)	102	(77.86)	195	(81.93)			
Previous psychological treatment for gambling							0.14	1	0.708
No	344	(93.22)	123	(93.89)	221	(92.86)			
Yes	25	(6.78)	8	(6.11)	17	(7.14)			
Tobacco use							0.61	1	0.468
No	61	(16.53)	19	(14.5)	43	(17.65)			
Yes	308	(83.47)	112	(85.5)	196	(82.35)			
Poly-consumption (excluding tobacco)							1.32	1	0.287
No	81	(21.95)	32	(24.43)	46	(19.33)			
Yes	288	(78.05)	99	(75.57)	192	(80.67)			

^a^df, degrees of freedom

### Attachment Style and Participant Characteristics

As shown in Table 3, none of the participant characteristics (i.e., sociodemographic variables, psychological treatment attendance, tobacco use, or poly-consumption) were significantly related to insecure attachment. However, participants with insecure attachment did have higher alexithymia and global severity index scores, as well as higher scores for all the psychological symptoms except somatization than participants with secure attachment (see Table 4).

**Table 4:** Differences in participant psychological variables by attachment style

			Insecure attachment					
	Total (n = 369)		No (n = 131)		Yes (n = 238)			
Variable	*M* ^*a*^	*SD* ^*b*^	*M*	*SD*	*M*	*SD*	*TW* ^*c*^	*p*
Alexithymia	59.75	10.87	55.61	11.56	61.49	10.21	-3.80	< 0.001
Global severity index	1.80	0.72	1.57	0.74	1.93	0.68	-4.36	< 0.001
Somatization	1.77	0.90	1.67	0.97	1.83	0.85	-1.75	0.080
Obsession-Compulsion	1.95	0.86	1.68	0.92	2.10	0.78	-4.26	< 0.001
Interpersonal Sensitivity	1.65	0.89	1.32	0.86	1.84	0.85	-5.49	< 0.001
Depression	2.29	0.85	2.08	0.93	2.40	0.79	-3.07	0.002
Anxiety	1.84	0.90	1.64	0.94	1.95	0.86	-3.22	0.001
Hostility	1.58	1.03	1.32	1.09	1.72	0.96	-4.18	< 0.001
Phobic Anxiety	1.12	0.91	0.82	0.78	1.29	0.93	-5.01	< 0.001
Paranoid Ideation	1.73	0.92	1.46	0.95	1.88	0.88	-4.18	< 0.001
Psychoticism	1.56	0.80	1.31	0.82	1.69	0.76	-4.47	< 0.001

^a^M, median

^b^SD, standard deviation

^c^TW, Wilcoxon non-parametric test

### Risk Factors for Problematic Gambling Behaviors

The multivariate logistic regression analysis indicated that sex, previous psychological treatment, and poly-consumption were related to problematic gambling (see Table 5). Men were nearly four and a half times more likely than women to have gambling problems. In addition, participants who consumed poly-substances and had no history psychological treatment had approximately three times the odds of having gambling problems than those who only consumed one substance or had history psychological treatment, respectively. Insecure attachment and alexithymia increased the probability of problematic gambling, with insecure attachment doubling the probability of gambling problems in comparison to the secure attachment style. The indirect effect of insecure attachment on the co-occurrence of gambling problems via alexithymia was significant (0.02; Bootstrap SE = 0.01, 95% CI Bootstrap [-0.052, -0.003]; z = -1.98, *p* = .048) and explained 17.04% of the variance. The regression model had a satisfactory discrimination level for the co-occurrence of problematic gambling behaviors based on the AUC. The Hosmer-Lemeshow test also confirmed that the model adequately fit the data.

**Table 5:** Multivariate logistic regression model for predicting the presence of gambling issues

		Regression coefficients	
Variable	Beta (SE)^a^	OR^b^ (95% CI^c^)	*p*
Sex			
Men	1.49 (0.93)	4.43 (2.26, 8.68)	< 0.001
Women	Reference	Reference	
Previous psychological treatment for substance abuse			
No	1.11 (0.33)	3.04 (1.58, 5.85)	< 0.001
Yes	Reference	Reference	
Poly-consumption			
No	Reference	Reference	
Yes	1.06 (0.40)	2.90 (1.33, 6.30)	0.007
Insecure attachment style			
No	Reference	Reference	
Yes	0.70 (0.29)	2.01 (1.14, 3.54)	0.016
Alexithymia^e^	0.03 (0.01)	1.03 (1.00, 1.05)	0.023
AUC^d^ (95% CI)		0.74 (0.68, 0.79)	
Hosmer-Lemeshow test		0.26	

^a^Beta (SE), Beta regression coefficients and their corresponding standard errors

^b^OR, odds ratio

^c^CI, confidence interval

^d^AUC, Area Under the Curve

^e^Increment per unit in this variable

## Discussion

This study investigated the presence of problematic gambling behaviors in individuals with substance use disorder and gathered information about psychological risk factors that could help explain gambling problems. Our research focused on the role of attachment styles in predicting problematic gambling behaviors in people with substance use disorder and the mediating role of alexithymia. Results suggest that insecure attachment is more common (and alexithymia scores are higher) in individuals with substance use disorder and problematic gambling than in those without gambling problems. Moreover, alexithymia was found to be a significant mediator in the relationship between an insecure attachment style and problematic gambling behaviors.

Our final model showed that men had a higher risk of gambling than women. This is in line with previous research regarding gambling problems in substance abuse and non-substance abuse contexts (Jiménez-Murcia et al., 2014; Savvidou et al., 2017). There was also an increase in the prevalence of poly-consumption in the study participants, which was related to a high risk of problematic gambling and highlights that men may be especially vulnerable (Jeffirs et al., 2019; Wu et al., 2011). In addition, previous psychological treatment for substance abuse was related to less probability of problematic gambling, which suggests that treatment for substance addiction may help prevent gambling problems (Rash et al., 2016). While psychological symptomatology has been strongly related to addiction (Jiménez-Murcia et al., 2009; Parhami et al., 2014; Rash et al., 2016), our results suggest that mental health was not a critical variable needed to explain gambling problems in individuals with substance use disorder. This may indicate that psychological disorders pose as common etiological factors for both addictions and do not increase the risk for the co-occurrence of gambling and substance abuse.

There was a higher prevalence of insecure attachment in participants with substance use and gambling problems than in those with substance use disorder alone. Previous research shown that people with gambling disorders (or at a high risk of gambling problems) have higher insecure attachment scores than those with no risk (Estévez et al., 2020; Kaya & Deveci, 2021). Furthermore, for those with problematic gambling behavior, insecure attachment also predicts the severity of gambling (Di Trani et al., 2017; Gori et al., 2022; Keough et al., 2018). These results indicate that people whose relationship with the caregiver was impaired during childhood increase their chance of addiction problems. In line with the self-regulation theory (Lafond Padykula & Conklin, 2010), the lack of a stable and safe bond with caregivers limits the opportunity exploration and learning adequate models of adaptation to the context. Consequently, the increased frequency of impaired attachment in individuals with substance use disorder and a gambling comorbidity may be due to difficulties in the emotional regulation and management of emotions and feelings that increase the risk of searching for other behaviors to self-regulate such are addictions. Our investigation extends knowledge gained from previous research and shows that insecure attachment is a risk factor for gambling and substance use disorders, even when controlling for poly-consumption, age and sex. Previous literature has suggested that attachment may influence different types of addictions (Schindler, 2019), and deficits in attachment appear to have a stronger relationship with behavioral as opposed to substance addictions (Estévez et al., 2017) such as alcohol, psycho-stimulants, or opiates. Our current results indicate that insecure attachment is also a greater risk factor for gambling than substance addictions in a clinical population with poly-consumption.

People with substance use disorders and problematic gambling behaviors had higher alexithymia scores than those without gambling problems. Increased alexithymia levels may act as mediators in the relationship between insecure attachment and gambling problems. Our results reinforce ideas from the literature, which highlight the importance of alexithymia in explaining attachment effects (Estévez et al., 2020; Sung et al., 2020; Toqeer et al., 2021). The role of alexithymia supports the relationship between insecure attachment and addiction due to problems in the self-regulation (Lafond Padykula & Conklin, 2010). Thus, the lack of stable and safe bonds to caregivers may lead to difficulties in acquiring adaptive strategies for emotion regulation. Concretely, the mediating function of alexithymia indicates that insecure attachment may facilitate poor identification and differentiation of emotions and feelings, problems describing feelings to others, and externally focused thinking (Di Trani et al., 2017; Estévez et al., 2020). Thus, alexithymia increases the emotional unrest as reduces the opportunity of properly handle emotions (Luminet et al., 2018). This emotional distress would then be alleviated by carrying out behaviors such as gambling that reduce this discomfort (Fletcher et al., 2015). As our results showed that this process is stronger for substance abusers with gambling problems, this research pinpoints towards the role of attachment and its impact on emotion regulation as a summative psychological factor for comorbid substance and behavioral addictions.

This study has implications for further research and therapeutic interventions. First, the high prevalence of problematic gambling behaviors in a clinical sample of individuals with substance use disorder highlights the importance of identifying the presence of comorbid gambling problems. To promote adequate interventions, future research should investigate the co-occurrence of behavioral addictions in individuals with substance use disorder to further our understanding of their underlying psychological processes. Second, due to the increased risk of gambling disorder linked to insecure attachment and alexithymia, interventions should integrate treatment for both issues to help individuals in their emotional self-regulation. As Liese et al., (2020) noticed, given the high stability of attachment, interventions might benefit by focusing on the promotion of adequate emotional regulation, for which cognitive-behavioral therapy and mindfulness interventions have proven beneficial.

This study has several limitations. First, the cross-sectional nature of the design does not allow for the testing of causal effects. Therefore, the present results should be viewed with caution. Future research should include longitudinal studies to test the reliability of the findings. Second, we measured insecure attachment and alexithymia as global variables; thus, our results do not provide information on the role of insecure attachment styles or different aspects of alexithymia on addiction. Consequently, future studies should investigate these facets of behavioral self-regulation to understand the complexity of the relationship between attachment and addictions.

## Conclusion

This study demonstrates that insecure attachment is an important risk factor for problematic gambling behaviors in individuals with substance use disorders. Moreover, alexithymia was shown to be an explanatory mechanism, which suggests that problems in emotion comprehension and mentalization can worsen the complexity of substance addiction with behavioral ones. These results provide novel information about the processes involved in the summative severity of substance addictions and offer clues for understanding and promoting interventions for individuals with these risk profiles.

## Electronic Supplementary Material

Below is the link to the electronic supplementary material.

Supplementary Material 1

## Data Availability

The data is on the Osakidetza’s (Public health system of the Basque Country) computer network, with computer security measures in accordance with the GDPR. The datasets generated during and/or analysed during the current study are available from the corresponding author on reasonable request.
