# Supplementary material for: Problematic Gambling Behavior in a Sample with Substance Use Disorder: The Role of Attachment Style and Alexithymia
Source: J Gambl Stud. 2022 Sep 24;39(2):513–29. doi: 10.1007/s10899-022-10154-2 (PMC10175442; doi:10.1007/s10899-022-10154-2)
Supplement: Supplementary file 1 — Supplementary Material 1 [file 10899_2022_10154_MOESM1_ESM.docx]

March 21st, 2022

Professor Jon Grant

Editor, ***Journal of Gambling Studies***

Dear Professor Grant,

Urko Aguirre-Larracoechea, Marta Herrero, Ana Estévez and I would like to have our paper entitled, “**PROBLEMATIC GAMBLING BEHAVIOR IN A SAMPLE WITH SUBSTANCE USE DISORDER: THE ROLE OF ATTACHMENT STYLE AND ALEXITHYMIA**” to be reviewed for possible publication in the ***Journal of Gambling Studies.*** We have not submitted this paper to any other journal.

We suggest the following reviewers:

María Iciar Iruarrizaga Diez – Universidad Complutense de Madrid

<[iciariru@psi.ucm.es](mailto:iciariru@psi.ucm.es)>

Patricia Macia – Universidad del País Vasco

<patricia.macia@ehu.eus>

Ana Mª Pérez García – Universidad Nacional de Educación a Distancia

<[aperez@psi.uned.es](mailto:aperez@psi.uned.es)>

Laiene Olabarrieta Landa – Universidad de Navarra

<[laiene.olabarrieta@unavarra.es](mailto:laiene.olabarrieta@unavarra.es)>

Jiménez Murcia, Susana – Belvitge Hospital

<[sjimenez@bellvitgehospital.cat](mailto:sjimenez@bellvitgehospital.cat)>

I will be serving as the corresponding author for this manuscript. The author listed in the byline has agreed to the byline order and to submission of the manuscript in this form.

Please let me know if you have questions or need additional information about our submission.

We greatly appreciate your consideration.

Respectfully Yours,

Mª Begoña Rueda Ruiz
